# Glances at the Hospitals

**Published:** 1900-08-04

**Authors:** 


### GLANCES AT THE HOSPITALS.

### THE ROTHERHAM HOSPITAL AND DISPENSARY.

The twenty-eighth annual report gives the statistics of the hospital for the year 1899, and shows that 4,874 patients received the benefits of the institution during that year. Of these 581 were in-patients, and 4,295 were out-patients. The average number of in-patients was 39, and the average duration of their stay in the hospital was 21 days. Two hundred and forty-three operations were performed, and, excepting in three cases, all were successful. The ordinary receipts amounted to £2,984, being about £57 above those for 1898. In addition to this, legacies amounting to upwards of £400 were received, and nearly £4,000 was received from the Mayoral Fund. Part of this sum will meet the cost of a new operating theatre, and £2,000 will be invested. It is noteworthy that the work people's subscriptions are considerably higher than in 1898. The honorary medical staff consists of Messrs. Baldwin, Lyth, Knight and Reid. Messrs. Martin and Bennett are house surgeons. Miss Sanders is matron.

### THE WHITWORTH HOSPITAL AT DARLEY DALE.

The report for 1899 is published as the first annual report of the Whitworth Hospital, founded by the devizees of the late Sir Joseph Whitworth, Bart., 1889, and re-opened by the Whitworth Hospital Trustees on 1st January, 1899. Patients are admitted from any district within a radius of eight miles from the hospital. It is maintained partly by endowments, but a considerble sum must be raised annually to keep it going, and the public subscribed £219. Patients contributed £29, and the payments ranged from 1s. to 7s. 6d. a week. Altogether 74 patients were treated during the year. These included 15 cases of typhoid fever, and 13 accident cases. Twenty-one operations were performed, and all were successful. The greater number of the cases have been acute and severe; the chronic element has not entered much. The honorary medical and surgical staff consists of Drs. Thorburn, Wrench, Mox n, Sharpe and Orme. Miss Hoyle is matron, and Mr. Dawson, secretary.

### THE STAFFORDSHIRE GENERAL INFIRMARY AT STAFFORD.

The total income for the year 1899 was £2,982, and the total expenditure was £2,752, showing a balance in favour of the institution of £230; but this balance was due to the transfer of £600 from the capital to the general account. There were 50 patients in residence at the beginning of the year, and 755 were admitted during the year, making a total of 805 under treatment. Of these, 397 were discharged cured, 188 convalescents were made out-patients, 120 were relieved, 5 were incurable, 31 died, 16 were discharged for various reasons, and 48 were under treatment on the date of this report. In the out-patients' department 1,360 cases were treated, in addition to 1,210 casualties and 987 dental cases, giving a grand total of 4,362 cases. There was an increase of 15 in-patients over those of 1898, and an increase of 122 out-patients. As regards the latter, we should much prefer to have been able to chronicle a decrease, and we venture to hope that a careful scrutiny will enable the Governors to effect some reduction during the current year. The average residence of in-patients was three weeks and four days, and the average expense per patient was nearly £3 4s., the daily average cost of each in-patient being 2s. 6¾d. An analytical table of diseases is given, and from it we learn that something like 150 distinct diseases were treated during the year, but the results of the treatment are not stated. It is to be desired that the next annual report of the Infirmary will enlighten us on this point, and will also give the results of the major operations. The medical staff consists of Drs. Reid, Greaves, C. Reid, Blumer, and Marson. Dr. Bonis is house surgeon, Dr. Gibson assistant house surgeon, Mr. Battle is secretary, and Miss Farmer is matron.

